# Hemostasis, myocardial protection, and advanced monitoring in cardiovascular anesthesia: evidence-based practical insights

**DOI:** 10.31744/einstein_journal/2025CE1982

**Published:** 2025-10-20

**Authors:** Alfredo Olivera Orellana

**Affiliations:** 1 Hospital Nacional Guillermo Almenara Irigoyen Lima Peru Cardiovascular Anesthesiologist, Hospital Nacional Guillermo Almenara Irigoyen, Lima, Peru.

Dear Editor,

I read with great interest the article by Vasconcelos et al., titled *"*Perioperative management of adult patients undergoing coronary artery bypass grafting and valve surgery: a literature review," recently published in einstein (São Paulo) (2025). The authors provide a comprehensive and up-to-date overview of anesthetic perioperative management in cardiac surgery, offering valuable guidance readily applicable to clinical practice.^(1)^

Based on their review and my experience as a cardiovascular anesthesiologist in Latin America, I would like to share four primary reflections that may help advance the discussion on perioperative care. These reflections focus on hemostasis, anesthetic techniques, intraoperative monitoring, and regional implementation challenges.

## RATIONAL USE OF TRANEXAMIC ACID

There is strong evidence supporting the use of tranexamic acid (TXA) to reduce bleeding and transfusion needs in cardiac surgery, even among patients receiving dual antiplatelet therapy.^(2,3)^ The OPTIMAL trial (2022), involving over 3,000 patients, demonstrated that high- and low-dose regimens had comparable efficacy, although high-dose TXA was associated with a higher risk of postoperative seizures.^(4)^

In practice, we favor a low-dose regimen of 10-15mg/kg as a bolus, followed by a continuous infusion of 1mg/kg/h. Recent systematic reviews support this safety-first approach, specifically for patients at higher neurological risk.^(5,6)^

## INDIVIDUALIZED PROTAMINE REVERSAL AFTER CARDIOPULMONARY BYPASS

I agree with the authors that protamine administration should be based on coagulation monitoring, such as activated clotting time (ACT), rather than on fixed empirical doses. Excess protamine may paradoxically impair coagulation, induce vasoplegia, and contribute to postoperative pulmonary dysfunction.^(7)^

At our center, we reverse heparin with 1-1.2mg protamine per 100IU of heparin, with adjustments based on real-time ACT values and clinical status.

## VOLATILE AGENTS VERSUS TOTAL INTRAVENOUS ANESTHESIA (TIVA): A CONTEXTUAL DECISION

Although the MYRIAD trial demonstrated no difference in long-term mortality between volatile anesthetics and TIVA, several studies highlight the cardioprotective effects of agents, such as sevoflurane, potentially associated with ischemic preconditioning.^(2,8)^ In the absence of remifentanil, TIVA using propofol alone may be less stable, specifically in patients with impaired left ventricular function.

We routinely use sevoflurane for valve and coronary surgeries, specifically in patients with left ventricular ejection fraction of <40%. For low-risk cases, propofol TIVA can be used under close hemodynamic monitoring.

## TRANSESOPHAGEAL ECHOCARDIOGRAPHY: A CORNERSTONE FOR SAFETY

Transesophageal echocardiography (TEE) provides real-time insights into cardiac function, volume status, and valvular dynamics, offering crucial support for intraoperative decision-making.^(9)^ However, its use remains limited in numerous Latin American institutions because of limited resources and formal training programs.

We propose incorporating structured TEE training into all cardiovascular anesthesia fellowships. In our department, we conduct biannual audits to assess the effect of intraoperative TEE on clinical outcomes, specifically in challenging cases, such as failed cardiopulmonary bypass weaning or unexplained hypotension.

## CONCLUSION

The study by Vasconcelos et al. offers a timely and relevant foundation for refining cardiovascular anesthesia protocols. Based on their findings and our experience, I recommend implementing rational low-dose tranexamic acid protocols, using individualized ACT-guided protamine reversal, continuing sevoflurane use when remifentanil is unavailable, and establishing formal transesophageal echocardiography training and audit systems.

These evidence-based and adaptable interventions can enhance perioperative safety and outcomes in cardiac surgery, specifically in resource-limited environments.

Sincerely,
